# Effects of simulated hypo-gravity on lower limb kinematic and electromyographic variables during anti-gravitational treadmill walking

**DOI:** 10.3389/fphys.2023.1141015

**Published:** 2023-06-08

**Authors:** Christopher A. Malaya, Pranav J. Parikh, Dean L. Smith, Arshia Riaz, Subhalakshmi Chandrasekaran, Charles S. Layne

**Affiliations:** ^1^ Center for Neuromotor and Biomechanics Research, Department of Health and Human Performance, University of Houston, Houston, TX, United States; ^2^ Grail Laboratory, Parker University, Dallas, TX, United States; ^3^ Nutrition and Health, Department of Kinesiology, Miami University, Oxford, OH, United States

**Keywords:** gait, hypogravity, unloading, hysteresis, kinematics, EMG, anti-gravity treadmill

## Abstract

**Introduction:** This study investigated kinematic and EMG changes in gait across simulated gravitational unloading levels between 100% and 20% of normal body weight. This study sought to identify if each level of unloading elicited consistent changes—particular to that percentage of normal body weight—or if the changes seen with unloading could be influenced by the previous level(s) of unloading.

**Methods:** 15 healthy adult participants (26.3 ± 2.5 years; 53% female) walked in an Alter-G anti-gravity treadmill unloading system (mean speed: 1.49 ±0.37 mph) for 1 min each at 100%, 80%, 60%, 40% and 20% of normal body weight, before loading back to 100% in reverse order. Lower-body kinematic data were captured by inertial measurement units, and EMG data were collected from the rectus femoris, biceps femoris, medial gastrocnemius, and anterior tibialis. Data were compared across like levels of load using repeated measures ANOVA and statistical parametric mapping. Difference waveforms for adjacent levels were created to examine the rate of change between different unloading levels.

**Results:** This study found hip, knee, and ankle kinematics as well as activity in the rectus femoris, and medial gastrocnemius were significantly different at the same level of unloading, having arrived from a higher, or lower level of unloading. There were no significant changes in the kinematic difference waveforms, however the waveform representing the change in EMG between 100% and 80% load was significantly different from all other levels.

**Discussion:** This study found that body weight unloading from 100% to 20% elicited distinct responses in the medial gastrocnemius, as well as partly in the rectus femoris. Hip, knee, and ankle kinematics were also affected differentially by loading and unloading, especially at 40% of normal body weight. These findings suggest the previous level of gravitational load is an important factor to consider in determining kinematic and EMG responses to the current level during loading and unloading below standard g. Similarly, the rate of change in kinematics from 100% to 20% appears to be linear, while the rate of change in EMG was non-linear. This is of particular interest, as it suggests that kinematic and EMG measures decouple with unloading and may react to unloading uniquely.

## Introduction

Previous work has demonstrated that human proprioception diminishes in hypogravity; limb matching tasks are less effectively completed (Lackner and DiZio, 1992) as well as approximations of limb position (Bringoux et al., 2012; Young et al., 1993). Mouchnino et al. (1996) found that anticipatory postural adjustments were notably absent below standard Earth gravity. Other studies have also found decreases in illusory kinesthetic responses to vibration (Lackner and DiZio, 1992; Roll et al., 1998) as well as significant differences in cortical waveforms and transmission in hypogravity (Mouchnino et al., 2017; Saradjian et al., 2013). These findings suggest pervasive alterations (both central and peripheral) to proprioception in response to hypogravity.

Hysteresis is the dependency of a system on its previous states, or history. In humans, hysteretic influences have been found not only at a cellular—receptor-based—level (Villalba-Gabea and Chiem, 2020; Villalba-Gabea, 2016; Xiao et al., 2010; Wei et al., 1986a; Wei et al., 1986b), but also in brain networks during transitions between consciousness and unconsciousness (Kim et al., 2018), as well as in the human visual and somatosensory cortices (Sayal et al., 2020; Prud’homme & Kalaska, 1994).

Wei et al. (1986a), Wei et al. (1986b) provided early evidence of hysteresis in muscle spindle receptors. In a series of studies examining the spindle outputs of the ankle musculature in anesthetized cats across a variety of joint angles, neural outputs were strongly influenced by whether stimulation had been increasing or decreasing, even at similar angles. These effects, however, extend beyond the scale of individual receptors. Prud’homme & Kalaska (1994) demonstrated the influence of hysteresis in primate proprioception—even further localizing these changes to the primary somatosensory cortex—during reaching tasks. Subsequent studies have extended these findings to include human joint position sense (Artz et al., 2015; Weiler and Awiszos, 2000).

In insects, the selection of gait type is sensitive to different locomotion speeds, and dependent on the direction of change (Fujiki et al., 2013). Despite the wide variety of morphologies, similar effects have also been examined in ostriches, dogs, horses and, notably, humans (Thortensson & Roberthson, 1987; Mohler et al., 2007; Aoi et al., 2011; Aoi et al., 2013; Abdolvahab & Carello, 2015; Daley et al., 2016). This dependency on previous states, then, appears to be a ubiquitous factor in locomotion under standard Earth-like conditions in both quadrupeds and bipeds.

However, the exact mechanisms behind these changes are not yet well understood. In particular, it is unclear what exact factors drive these changes and if these changes are borne primarily of internal responses, external stimuli, or—more likely—some combination of both. To examine this, previous investigations have focused on manipulating the gait characteristics of an individual within an Earth-like environment. Few studies have sought to examine hysteresis in human gait through manipulation of the environment itself (Bringoux et al., 2012; Ivanenko et al., 2002; Young et al., 1993). Yet even a brief consideration of the ontology of gait reveals the absolute importance of environmental conditions to human locomotion.

In this study, we sought to manipulate the environment surrounding human gait; specifically, we simulated reducing gravitational conditions for healthy adults in order to investigate the effects of gravity as a driving force for hysteretic changes during treadmill walking with loading and unloading. This study has important ramifications in the rehabilitation of lower extremity injuries, where loading and unloading are common parameters of therapy (Ülger et al., 2018; Łyp et al., 2016; Tanaka et al., 2013).

These questions were addressed through the use of zero-dimensional (traditional kinematic and electromyographic measures) and one-dimensional (utilizing statistical parametric mapping) analyses. Previous use of these methods has been found to provide complementary information that was not otherwise apparent given use of only one or the other (Layne et al., 2022a; Layne et al., 2022b).

## Materials and methods

### Participants

The participants in this study were 15 healthy adults (26.3 ± 2.5 years; 65.5 ± 4.7 inches; 151.7 ± 36.8 lbs; 53% female). Participants also did not have a history of, or any current systemic, degenerative or neuromusculoskeletal injuries or disease that could affect their ability to walk with differential loading for 15 min.

### Experimental protocol

#### Kinematic sensors

Participants were fitted with seven XSens (XSens Technologies) inertial measurement units (IMUs) arranged in a lower-body configuration. These sensors were placed bilaterally over the insteps of the feet, as well as anteriorly over the tibia at mid-shank and laterally over the mid-thigh. The final sensor was placed over the sacrum, centered at the S2 tubercle. All XSens sensors were secured by proprietary neoprene straps with non-slip, rubber backings.

#### Electromyographic sensors

Four dry surface electromyographic sensors (Biometrics Ltd.—model SX230) were adhered—using hypo-allergenic, double-sided tape—over the rectus femoris, biceps femoris, medial gastrocnemius and tibialis anterior of the right lower limb. These sensors were placed over the belly of each respective muscle—conduction surfaces in line with the muscle fibers—after the skin was shaved and scrubbed with an alcohol wipe. The electromyography control unit was held against the lower back of each participant by an elastic, Velcro-secured neoprene waistband.

#### Unloading system and walking protocol

Participants were asked to wear a pair of AlterG (AlterG Inc.) compatible neoprene shorts over their clothing. These shorts are designed to allow the participant to be secured into an AlterG Unloading Treadmill System, and a shell surrounding the treadmill system to inflate, thereby creating a positive pressure environment. This positive pressure environment can be used to reduce participants’ effective weight.

After being fitted into the system, participants were asked to walk at 100% normal loading for 5 min at a self-selected, comfortable speed that they could easily maintain for at least 15 min (mean speed: 1.49 ±0.37 mph). This allowed participants to become familiar with the system and allowed time for their gait to stabilize. Participants were also instructed not to hold onto the stability bars of the Alter-G system, but to allow their arms to swing normally. After the five-minute acclimation period, participants were unloaded to 20% of their body weight, in 20% increments, spending one full minute at each level during the descent. After completing one minute of walking at 20%, the protocol was reversed; participants walked for one minute at 40%, 60%, 80% and 100% of their body weight, respectively and in that order. For all levels of unloading and loading, the treadmill speed remained at the participants previously self-selected speed. Participants underwent this pyramidal design of unloading and loading with immediate movement to the next level in the protocol (i.e., no rest or quiet stance in between levels). Kinematics and electromyography data were recorded for the final minute of the acclimatization period, as well as the full minute of walking at all levels of unloading and loading. The reduction in load required approximately 10 s during which time data was not collected.

#### Data processing

Kinematic data were streamed wirelessly from the XSens IMUs to a computer running a data collection software suite (MVN Awinda). This software collected and internally calculated joint angles for the hip, knee, and ankle, bilaterally. Joint angle waveforms were separated into strides and normalized to 100 points using the peak knee as a reference. Mean, maximum and minimum angles were extracted for all joints. Data were exported, organized, and statistically analyzed in MATLAB using custom scripting.

Electromyographic (EMG) data were collected by a surface EMG system (Biometrics Ltd.). Four channels of data were simultaneously recorded by a waist-mounted control unit, as well as streamed to a computer running a data collection software suite (DataLOG). Data collected were exported into MATLAB for processing. Each channel was individually bandpass filtered (20–450 Hz) using a 2nd order Butterworth filter. Waveforms were then full wave rectified and enveloped using a low pass filter with an additional 2nd order Butterworth filter utilizing a cutoff frequency of 40 Hz (Winter et al., 1980). EMG data were separated into strides and normalized to 100 points using the kinematic peak knee timestamps as a reference. After processing, peak amplitude values, root-mean-square (RMS) and integrated areas were calculated for all muscles. RMS was calculated as the square root of the mean of all values squared over the entire time interval at each level of loading, as a measure of the amplitude of the EMG signal (Cifrek et al., 2009). Integrated areas were also collected over full time intervals and represent the total electrical signal or drive from the central nervous system to the motorneuron (Carpentier et al., 2001; Barton and Hayes, 1996; van der Hoeven et al., 1993; Linssen et al., 1993; Enoka, 1988).

Difference waveforms were also created for both kinematic and electromyographic waveforms. Adjacent kinematic and electromyographic waveforms for the hip, knee and ankle were subtracted from their nearest neighbor (e.g., 100% load—80% load; 80%–60% load, etc.) creating four total waveforms per joint and muscle. These resulting waveforms represent the distance between two adjacent waveforms (e.g., 100% load, and 80% load) and thus, when compared, offer insight into the linearity of change between levels of load.

Phase diagram and angle-angle diagrams were also created for the hip, knee, and ankle joints in order to compare both the coordination and movement strategies employed at 100% and 20% loading.

#### Statistical analysis

Kolmogorov-Smirnov and Shapiro-Wilk tests revealed all data were normally distributed and Mauchly’s test showed sphericity was preserved.

#### Zero-dimensional analysis

Kinematic and EMG variables were tested for normality and sphericity using the Kolmogorov-Smirnov and Shapiro-Wilk tests, as well as Mauchly’s test, respectively. Mean, maximum and minimum angles and range of motion (ROM), for each joint, as well as peak value, RMS and integrated areas were compared across all levels of loading using repeated measure ANOVAs. Post hoc testing was performed with corrected, paired t-tests, as appropriate.

#### One-dimensional analysis

Differences waveforms for kinematic and EMG data were compared across all levels of loading utilizing SPM f-tests. Post hoc testing was performed with individual SPM paired t-tests, as appropriate.

## Results

### Kinematics

**FIGURE 1:**
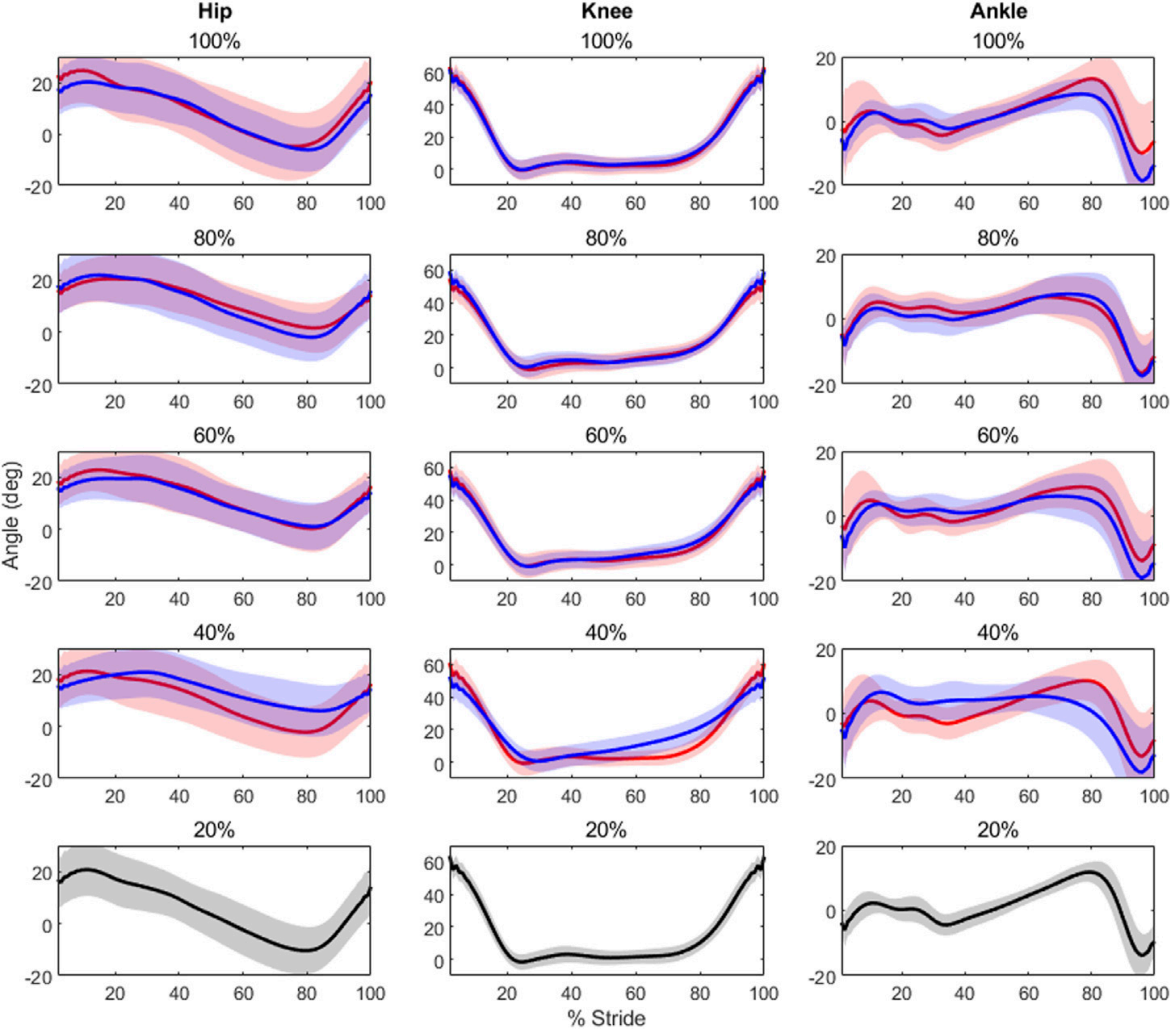
Each plot contains the average kinematic waveforms for its respective unloading (in red) and loading (in blue) condition, along with a 2-standard deviation shaded area around each waveform. All 20% load conditions are in black to avoid any confusion, as only a single waveform is present. Note the low variability of the knee waveforms across loading levels, and irrespective of absolute level of load. Conversely, the ankle shows higher variability between like levels, though it remains similar across absolute levels of load.

Results showed that level of unloading had a statistically significantly effect on hip mean [F(8,112) = 2.354, *p* = 0.0223], minimum [F(8,112) = 7.960, *p*< 0.0000] and ROM [F(8,112) = 12.2, *p*< 0.0000] values; knee mean [F(8,112) = 2.159, *p* = 0.0359], maximum [F(8,112) = 30.711, *p*< 0.0000] and ROM [F(8,112) = 8.34, *p*< 0.0000] values; as well as ankle maximum [F(8,112) = 6.649, *p*< 0.0000] and minimum values [F(8,112) = 3.692, *p* = 0.0007]. Post-hoc testing results are displayed in Table 1, and average kinematic waveforms by level of load are displayed in Figure 1.

**TABLE 1:** All comparisons in which *p* < 0.05 are presented. * denotes significance after correction for multiple comparisons. U denotes decreasing load (i.e., 40% load down from 60%); L denotes increasing load (i.e., 60% load up from 40%). Zero-dimensional kinematic pairwise testing.

	Measure	Level of unload	μ°±std	*p*-value
Hip	*ROM*	80U	19.9 ± 6.2	*p*< 0.0000*
		80L	24.9 ± 5.4	
		40U	25.0 ± 4.8	*p* = 0.0001*
		40L	17.1 ± 5.1	
	*Min*	40U	−2.8 ± 9.9	*p* = 0.0009*
		40L	4.3 ± 8.4	
Knee	*ROM*	80U	56.1 ± 6.5	*p*< 0.0000*
		80L	59.8 ± 6.5	
		60U	60.5 ± 7.1	*p*< 0.0000*
		60L	56.7 ± 6.3	
		40U	63.4 ± 7.7	*p*< 0.0000*
		40L	52.4 ± 7.0	
	*Mean*	40U	15.7 ± 5.2	*p* = 0.0295
		40L	18.6 ± 6.4	
	*Max*	80U	54.6 ± 8.2	*p*< 0.0000*
		80L	59.2 ± 6.9	
		60U	58.5 ± 9.1	*p* = 0.0008*
		60L	55.4 ± 7.9	
		40U	60.9 ± 8.8	*p*< 0.0000*
		40L	52.5 ± 9.7	
	*Min*	40U	−2.4 ± 5.7	*p* = 0.0314
		40L	0.1 ± 6.5	
Ankle	*Max*	100U	14.8 ± 7.5	*p* = 0.0020*
		100L	9.5 ± 3.8	
		60U	11.4 ± 7.4	*p* = 0.0332
		60L	8.6 ± 4.3	
	*Min*	100U	−14.0 ± 9.0	*p* = 0.0004*
		100L	−19.4 ± 8.5	
		60U	−16.9 ± 10.3	*p* = 0.0379
		60L	−19.8 ± 11.8	

### Electromyography

**FIGURE 2:**
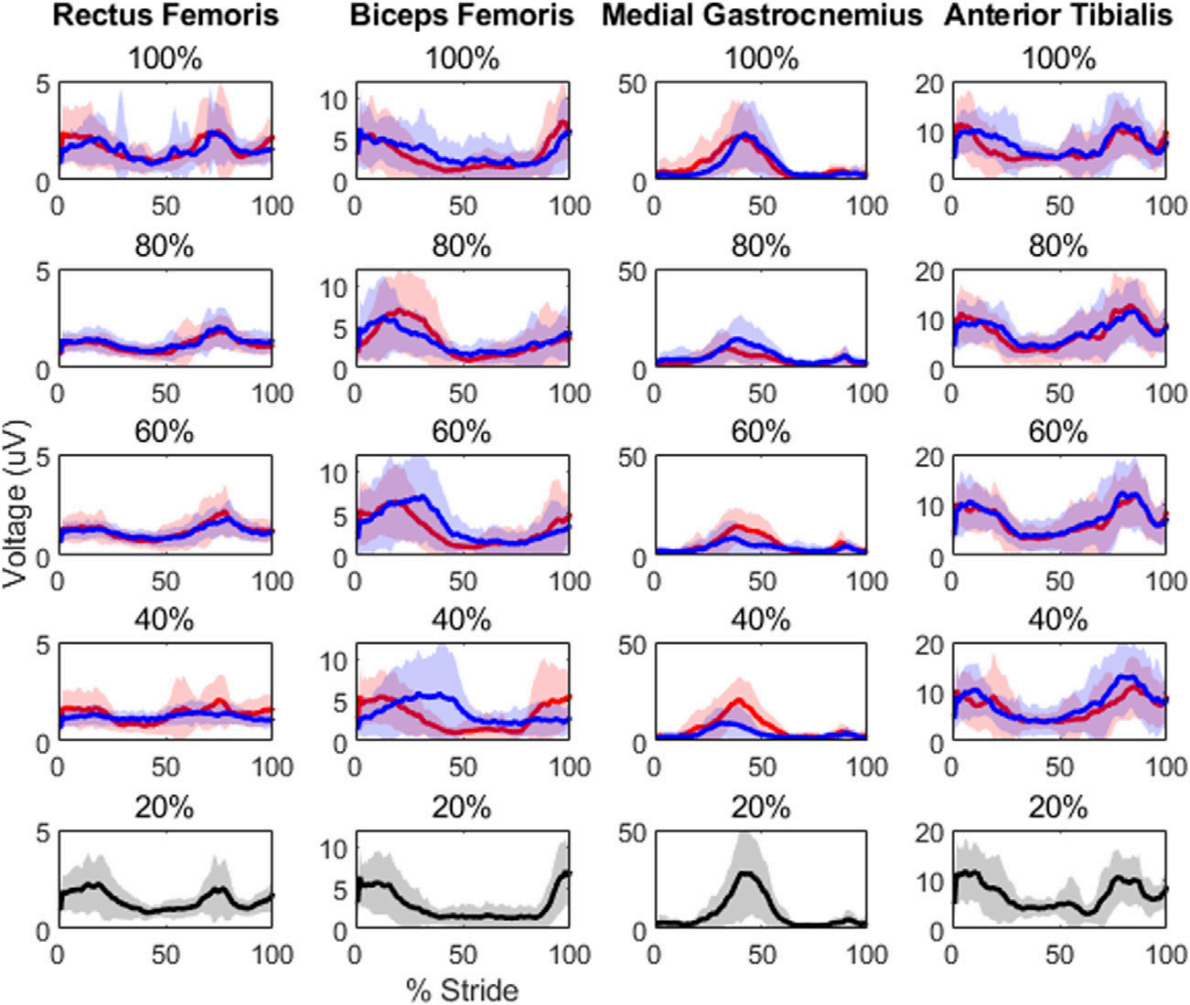
Each plot contains the average EMG waveforms for its respective unloading (in red) and loading (in blue) condition, along with a 2-standard deviation shaded area around each waveform. All 20% load conditions are in black to avoid any confusion, as only a single waveform is present. Though the phasic properties of these muscles appear to be robust with unloading, note the clear peak differences in the medial gastrocnemius as well as in the rectus femoris at 40% load depending on whether participants were loaded or unloaded previously.

Results showed level of unloading had a statistically significant effect on peak muscle activity in the rectus femoris [F(8,126) = 3.1, *p* = 0.0032] and medial gastrocnemius [F(8,126) = 4.72, *p*< 0.0000]. Root-mean-square and integrated area values were also statistically significant in the medial gastrocnemius [F(8,126) = 5.87, *p*< 0.0000; F(8,126) = 6.21, p< 0.0000, respectively]. There were also significant differences in the rectus femoris [F(3,42) = 5.3959, *p* = 0.0031] and medial gastrocnemius muscle waveforms across levels of load [F(3,42) = 21.3502, *p*< 0.0000]. Post-hoc testing results are displayed in Tables 2, 3 and average EMG waveforms by level of load are displayed in Figure 2.

**TABLE 2:** All comparisons in which *p* < 0.05 are presented. * denotes significance after correction for multiple comparisons. U denotes decreasing load (i.e., 40% load down from 60%); L denotes increasing load (i.e., 60% load up from 40%). Zero-dimensional EMG pairwise testing.

	Measure	Level of unload	$\mu \;\left(mV\right)\;\pm std$	*p*-value
Rectus Femoris	*Peak*	100U	4.4646 ± 2.7421	*p* = 0.0306
		100L	3.9922 ± 2.6994	
		60U	3.2659 ± 1.2942	*p* = 0.0148
		60L	2.5617 ± 0.7725	
		40U	3.7457 ± 1.5659	*p* = 0.0012*
		40L	2.0757 ± 0.5369	
Medial Gastrocnemius	*Peak*	60U	21.2675 ± 7.6419	*p*< 0.0000*
		60L	14.7524 ± 8.5479	
		40U	26.6887 ± 11.8060	*p* = 0.0006*
		40L	15.7406 ± 9.4869	
	*RMS*	60U	7.7948 ± 3.4918	*p* = 0.0002*
		60L	5.2989 ± 3.2454	
		40U	9.9422 ± 3.9645	*p*< 0.0000*
		40L	5.2791 ± 2.9565	
	*Integrated Area*	100U	779.2357 ± 325.6160	*p* = 0.0433
		100L	677.7923 ± 308.9550	
		80U	392.8107 ± 179.3109	*p* = 0.0006*
		80L	581.6962 ± 230.9010	
		60U	527.7233 ± 224.8672	*p* = 0.0003*
		60L	374.6759 ± 209.7692	
		40U	642.2513 ± 217.69.67	*p*< 0.0000*
		40L	358.9621 ± 160.8824	

**TABLE 3:** * denotes significance after correction for multiple comparisons. U denotes decreasing load (i.e., 40% load down from 60%); L denotes increasing load (i.e., 60% load up from 40%). One-dimensional EMG pairwise testing.

	Level of unload	Comparators	*p*-value
Rectus Femoris	100/80	80/60	*p* = 0.0011*
		60/40	*p* = 0.0001*
		40/20	*p* = 0.0011*
Medial Gastrocnemius	100/80	80/60	*p* = 0.0050*
		60/40	*p* = 0.0002*
		40/20	*p* = 0.0000*

### Post-hoc testing by percentage of body weight

The following percentage changes are calculated with the unloading condition as reference.

### 100% body weight

Average ankle joint angle maximum values decreased by 35.8% (14.8 ° to 9.5 ° ; *p* = 0.0020). Average angle joint angle minimum values decreased by 39.3% (−14.0 ° to −19.4 ° ; *p* = 0.0004).

### 80% body weight

Average knee maximum joint angles increased by 8.42% (54.6 ° to 59.2 ° ; *p*< 0.0000). Average hip ROM increased by 25% (19.9 ° to 24.9 ° ; *p*< 0.0000). Average knee ROM increased by 6.6% (56.1 ° to 59.8 ° ; *p*< 0.0000).

Average integrated area of the medial gastrocnemius increased by 48.1% (392.8107–581.6962 *mV*; *p* = 0.0006).

### 60% body weight

Average knee maximum joint angles decreased by 5.2% (58.5 ° to 55.4 ° ; *p* = 0.0008). Average knee ROM decreased by 6.2% (60.5 ° to 56.7 ° ; *p*< 0.0000).

Average peak muscle activity in the medial gastrocnemius decreased by 30.6% (21.2675–14.7524 *mV*; p< 0.0000). Medial gastrocnemius RMS decreased by 32% (7.7948–5.2989 *mV*; *p* = 0.0002). Average integrated area of the medial gastrocnemius decreased by 28.8% (527.7233–374.6759 *mV*; *p* = 0.0003).

### 40% body weight

Average hip minimum joint angles increased by 253% (−2.8 ° to 4.3 ° **;** *p* = 0.0009). Average hip ROM decreased by 31.6% (25 ° to 17.1 ° ) from unloading to loading (*p* = 0.0001). Average knee maximum joint angles increased 13.8% (60.9 ° to 52.5 ° ; p< 0.0000). Average knee ROM decreased by 17.3% (63.4 ° to 52.4 ° ; p< 0.0000).

Average peak muscle activity in the rectus femoris decreased by 44.5% (3.7457–2.0757 *mV*; *p* = 0.0012). Average peak muscle activity in the medial gastrocnemius decreased by 41% (26.6887–15.7406 *mV;* p< 0.0006). Medial gastrocnemius RMS also decreased, dropping 47% (9.9422–5.2791 *mV*; *p*< 0.0000). Average integrated area of the medial gastrocnemius decreased by 44.1% (642.2513–358.9621 *mV*; *p*< 0.0000).

## Discussion

This study investigated the kinematic and EMG changes in human gait across different levels of simulated gravitational unloading between 100% and 20% of normal body weight. It specifically sought to identify if each level of unloading elicited robust, consistent changes—particular to that percentage of normal body weight—or if the changes seen with unloading could be influenced by the previous level(s) of unloading. We found that hip, knee, and ankle kinematics as well as electromyographic (EMG) activity in the rectus femoris, and medial gastrocnemius were significantly different at the same level of unloading, having arrived from a higher, or lower level of unloading, respectively. Similarly, the rate of change in kinematics from 100% of normal body weight, down to 20% appears to be linear, as evidenced by the lack of significance in difference waveforms between these levels; however, significant disparities in rectus femoris and medial gastrocnemius electromyographic difference waveforms suggest that the differences seen in EMG data between 100% and 80% load are not the same as those found between 80% and 60%, 60% and 40%, and 40% and 20%. This is of particular interest, as it suggests that kinematic and EMG measures decouple with unloading and may react to unloading uniquely.

The results of this study provide additional evidence that kinematic and electromyographic features do not scale across load levels proportionally with each other (Cappellini et al., 2006; Ivanenko et al., 2002). While it possible to accurately predict muscle activity from kinematics alone (Manzano and Serrancoli, 2021), work by Mauntel et al. (2017) did find kinematic-EMG decoupling depending on the type of movement being performed. In that way, the findings of this study—that kinematics scale linearly down to 20% of body weight, while muscle activity displays non-linear scaling as weight is decreased similarly—are not particularly surprising. Indeed, this suggests that muscle activity may be a more sensitive responder to load, whereas joint angles and coordination may be more robust to changes in levels of loading. This is supported by the phase diagrams comparing 100%–20% load (see Figure 5). Similarly, the overall reductions in hip, knee, and ankle ROM as well as general reductions in muscle activity of the medial gastrocnemius are consistent with previous work examining unloaded gait (Apte et al., 2018; Awai et al., 2017). However, at 80% load, this study found that the maximum angles of the knee as well as ROM of both the hip and knee increased, alongside overall muscle activity of the medial gastrocnemius. These findings contrast with established literature, but potentially provide insight into participant responses to this unloading paradigm. All of the participants in this experiment were unloading-naïve, having never walked or run in an unloading system nor experienced unloading in any other scenario. As such, two possible explanations for the increase in medial gastrocnemius activity and ROM are due to the novelty of the unloading treadmill and/or the unloading environment. However, as participants were given an acclimation period, it is unlikely that the novelty of treadmill system itself was the driving effector behind these alterations. Rather, it is possible these changes were exploratory strategies in response to the new unloading environment. This is also supported by our findings utilizing statistical parametric mapping (see Table 3), which found that the change in EMG waveforms for the rectus femoris and medial gastrocnemius between 100% load and 80% were significantly different from all other changes between levels. This suggests that the initial experience of unloading can drive gait alterations independently of the level of unload. Future work in this area should consider acclimation not only to the medium of locomotion (e.g., treadmill, overground) but to the experimental paradigm as well (i.e., unloading, re-loading).

**FIGURE 3:**
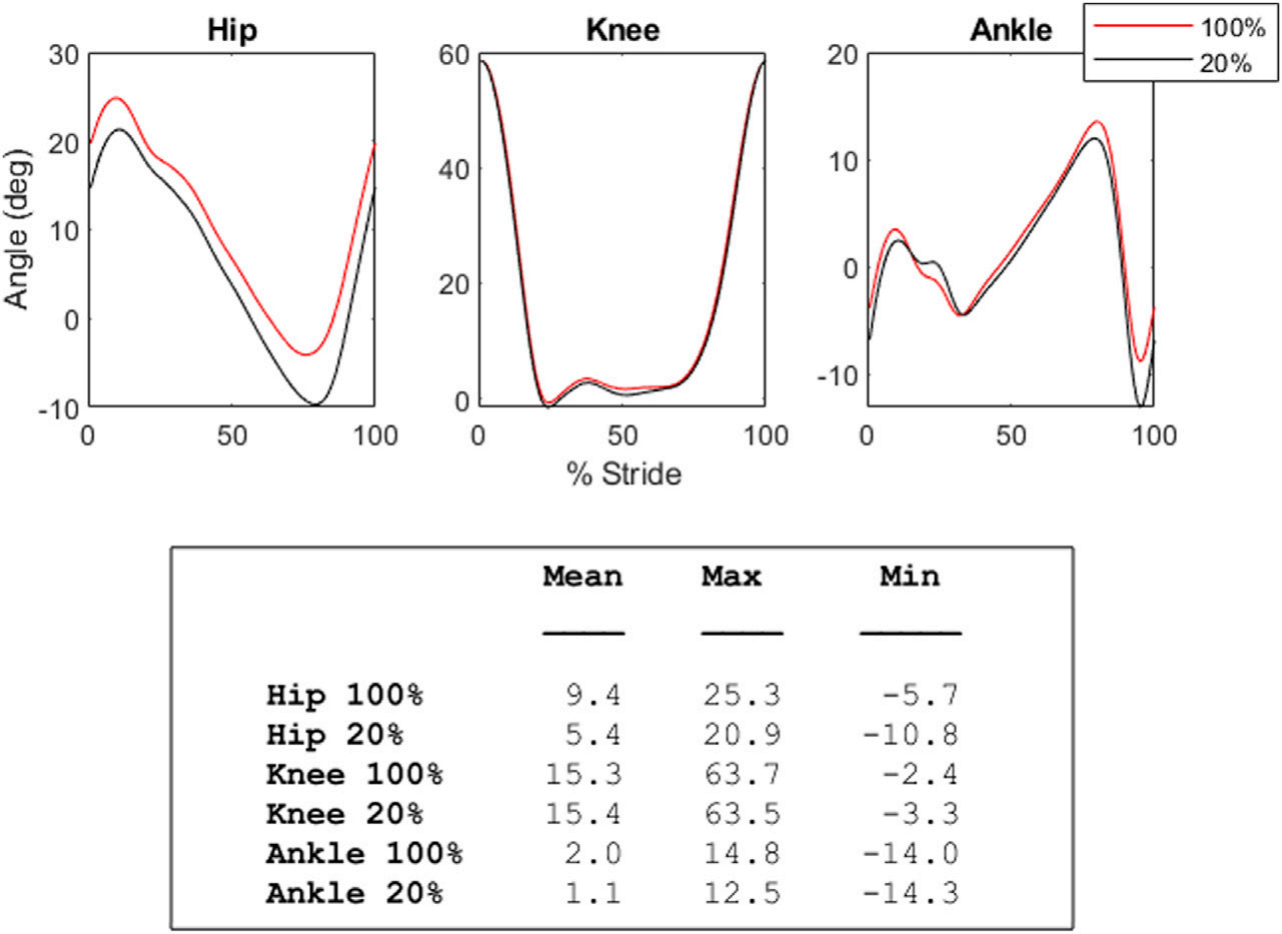
The graphs in this figure depict both the average 100% (baseline, red) and 20% (black) unloading conditions for the hip, knee, and ankle. In the hip, smaller joint angle values correspond with increased extension, while higher joint angle values correspond with increased flexion. In the knee, higher values are flexion, and lower are extension. In the ankle, higher values indicate plantar flexion, while lower values indicate dorsiflexion. The table below shows the mean, maximum, and minimum joint angle values (in degrees) for these conditions.

**FIGURE 4:**
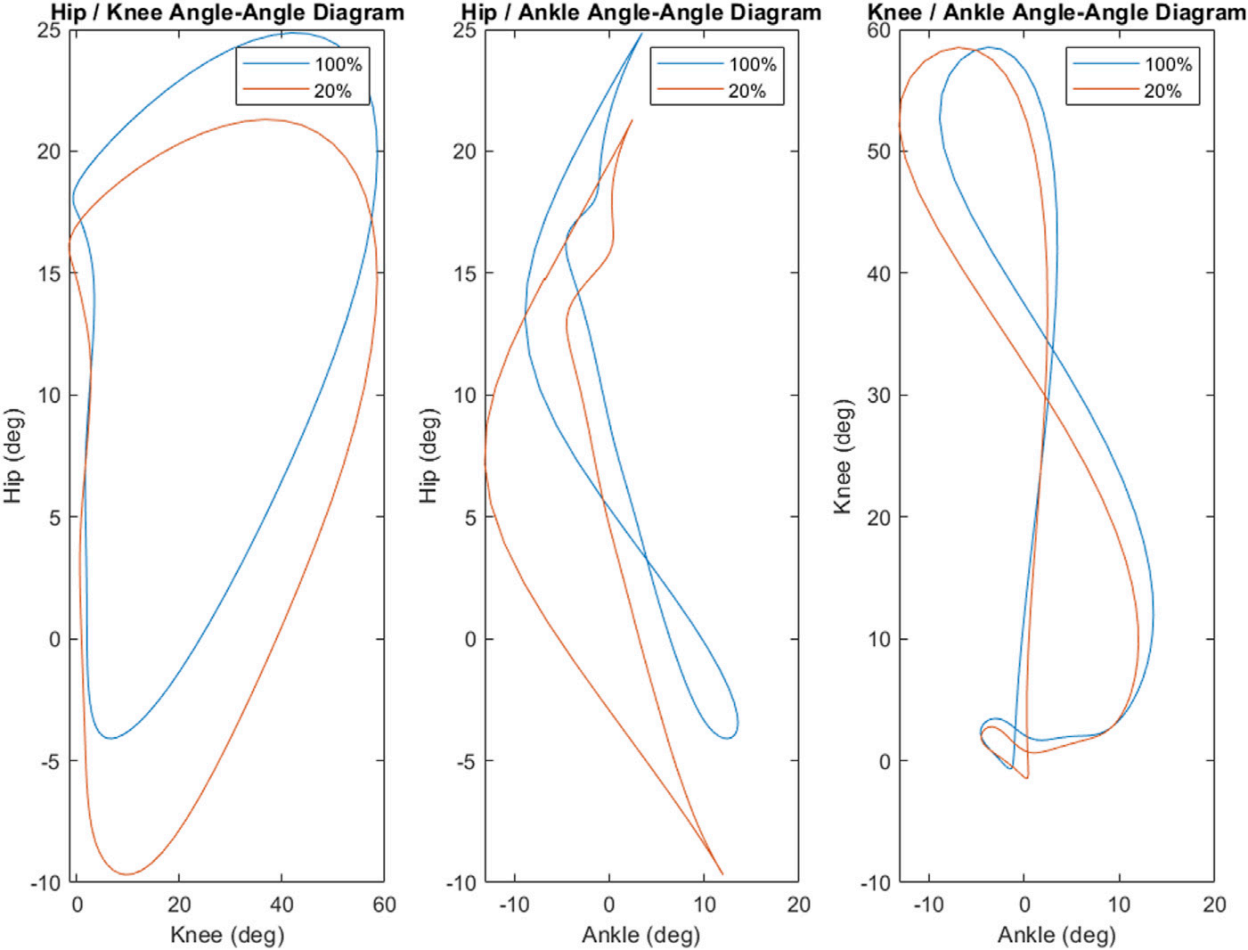
The coordination strategy between the joints of the lower extremities appears to be generally robust as load was decreased. However, there is clear stretching and translating in the coordinative strategies between the hip and knee as well as the hip and ankle. The hip and ankle, in particular, demonstrates a marked shift in coordinative strategy as the hip enters hyperextension. Coordination between the knee and ankle appears to be mostly preserved between 100% and 20% loads, though there is some stretching and shifting as load is decreased.

**FIGURE 5:**
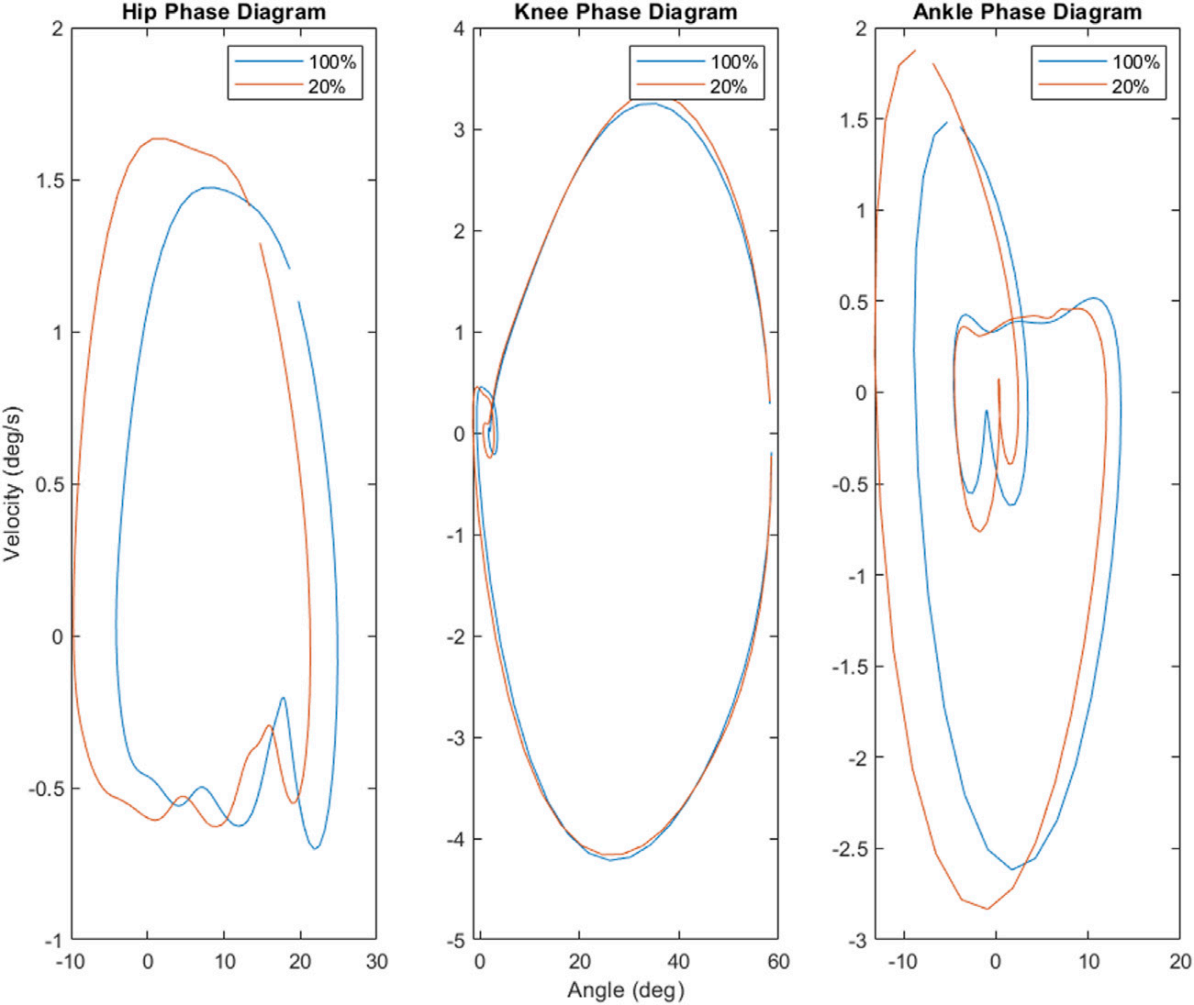
Phase portraits for the hip, knee and ankle suggest that the hip and ankle are most sensitive to shifts in load. Both the hip and ankle demonstrate notable expansion of the phase space (and thus possible states), while the available states of the knee are almost entirely unaffected by the decrease in load.

Phase diagrams (also known as phase portraits) are a graphical representation of a dynamic system in state space (Stergiou, 2003); more specifically, a phase portrait represents all of the possible behaviors of a system over a given time course. In this study, our phase portraits show the possible positions (the joint angle) and velocity (its rate of change) that a joint could inhabit over the course of single stride. An examination of the phase portraits between 100% and 20% load reveal a number of interesting details. The knee is arguably the most striking, revealing an almost perfect overlap between the two conditions (see Figure 5)—this is further supported by the clear lack of differences between the knee mean, maximum and minimum at these respective levels (see Figure 3). By contrast, the hip and ankle appear to translate at 20% of load, occupying similarly shaped phase spaces but in altered locations. That is, these similarly shaped phase spaces occur at different percentages of two waveforms. Considering the coordination between joints (as seen in Figure 4), it becomes apparent that the changes seen with unloading to 20% are driven primarily by coordinative changes in the hip and ankle, and, more specifically, the relationship between the two joints (Figure 4, middle panel). It is worth noting however, the robustness with which gait appears to scale; indeed, the coordinative relationships between joints of the lower extremities are mostly preserved, despite the relative change in joint angles.

While this study did not specifically quantify proprioception, the reduction in proprioceptive information at hypogravity is a likely effectual factor at work as our participants were unloaded. Interestingly, this study found that several equal levels of unloading were significantly different depending on whether the participants were being loaded (increasing weight) or unloaded (decreasing weight) to a given level. Work by Thiel et al. (2014) found that the strongest effects of hysteresis were found when sensory information was the weakest. Indeed, it is clear that perceptual judgements can be affected by the availability—or paucity—of information about an impending action (Abdolvahab & Carello, 2015).

As unloading decreases the amount of proprioceptive information, a possible explanation for the differences in equal loading levels is that they are not—in this case—environmentally different, but that the movement from less sensory information to more (loading), or more sensory information to less (unloading) invokes hysteretic changes. Put simply, as participants’ load increases, the relative utilization of environmental sensory information will drive their behavior more strongly than the previous level of load; inversely, as participants’ load decreases, the reduction of sensory information will facilitate the use of information from the previous level of loading. This is supported physiologically by work by Kostyukov & Cherkassky (1997) which found that discharge rates in spindles were always higher after stimulation rate increases, and, in fact, lower after decreases. Further, it appears that some of these effects are modulated through plantar pressure stimulation. Previous investigations of unloading have found that the removal of plantar support triggers reflexive decreases in slow-twitch muscle unit activity (Kozlovskaya et al., 2007). This in turn leads to rapid atony of extensor muscles with a potentially linked reductions in proprioceptor activity (Shenkman and Kozlovskaya 2018; Shenkman et al., 2017). Over longer periods of unloading than this study examined, this can lead to decreases in strength-speed properties, as well as changes in motor control (Saveko et al., 2022; Shpakov et al., 2008). Though interestingly, some of these alterations from unloading can be mitigated with plantar pressure stimulation (Litvinova et al., 2004).

Given this sensitivity of spindle receptors to changes in stimulation, gravitational changes—or more applicably here on Earth, weight—are likely a strong driving force behind the hysteretic changes seen in this study and others. This has far-reaching implications for a number of fields. In rehabilitation, loading and unloading cannot be considered equivalent activities, even if they are achieving the same loading conditions. In that way, increasing patient load could foster a greater reliance on the sensory information pertaining to the actual environment, whereas decreasing patient load would drive hysteretic changes in which the patient bases their response more fully on the previous level of load. This potentially allows for more targeted therapeutic interventions towards proprioceptive systems versus musculoskeletal effectors. Also, as there are marked kinematic and electromyographic changes at particular levels of load, providers should be cautious to ensure that patients are responding to the desired level of load, and not a previous one. Considering the hysteric changes seen in this study, moving from a level of lower loading to the (higher) desired level of load should ensure that patients are responding to the desired level—due to the relative abundance of sensory information - and not basing their gait on previous models and estimations.

It is important to note that the average comfortable speed selected by our participants $\left(1.49\;mph;\approx 0.67\frac{m}{s}\right)$ can approach walk-to-run and run-to-walk transitions as unloading increases. A study by Ackermann and van den Bogert (2012) found that at walking speeds of 1.1 $\frac{m}{s}$ , individuals sub-volitionally shifted to a bounding/skipping-type gait when unloaded to equivalent Moon gravity (1.63 $\frac{m}{s^{2}}$ , or about 16.6% of Earth gravitational acceleration), though participants did maintain a walking-style gait at Mars-level gravity (3.72 $\frac{m}{s^{2}}$ , ≈ 38% of Earth gravity). Given such, there appears to be a transition point in gait-type between these two levels, which contains our lowest unloading condition of 20% body weight. This could influence some of the hysteretic effects observed in this study at transitions to-and-from 20% of body weight. It is useful in this instance, however, to consider this issue in light of the dynamic similarity hypothesis (Alexander, 1976) and specifically, the Froude number. The Froude number (Fr) is a dimensionless parameter relating potential and kinetic energy given by the equation $Fr=\frac{v^{2}}{gh}$ , where v is the participants walking speed, g is the acceleration due to gravity and h is the height of the center of mass, approximated by leg length. Using our participant’s average selected walking speed (0.67 $\frac{m}{s}$ ), lowest gravitational acceleration of 1.962 $\frac{m}{s^{2}}$ (20% Earth gravity) and the expected Froude number associated with gait transitions of 0.5 (Alexander, 1989; Kram et al., 1997), we are left with $Fr=0.5=\frac{{\left(0.67\right)}^{2}}{\left(1.962\right)h}$ , which, solving for h equals 2.18 m. Clearly, the average participant in our study could not morphologically have a center of mass or equivalent leg length of 2.18 m. However, our fastest walking participant did achieve a comfortable speed of 0.89 $\frac{m}{s}$ , though they had a leg length of 90cm, which resulted in a Fr 0.44, and still below 0.5 at 20% load. Further, Figures 4, 5 present the average coordination waveform and phase diagrams utilized by participants for locomotion at 100% load and 20% load, and though there is definite shifting and stretching of these waveforms, they appear to maintain a robust walking-style shape, despite the unloading. With the above in mind, we feel comfortable that the effects of unloading and loading seen in this study are primarily due to hysteretic effects, rather than a gait shift. That being said, gait transitions could be important potential factors in hysteretic changes under reduced loads, and future work should consider addressing this.

Although admittedly speculative, given the changes in coordination and kinematics with unloading observed in this study, there are potential implications for the development of spacesuits. In previous, unpublished work, we found the switch to bounding gait did not occur until very close to 20% of load. With notably different gravitational conditions found on the Earth, its Moon as well as Mars, a single spacesuit designed with any of the single environments in mind may not be able to accommodate the others efficiently and could lead to stress and injury if applied in the wrong environment. Further, designers should be considerate of the previous level of gravitation that users have experienced, as this can influence even responses in the current one. In that way, it may be more effective to design spacesuits with a variable ability to support astronaut’s body weight. This would allow users to be unloaded below the target level of gravitation, before being loaded up to the target. This would help ensure the user was biomechanically responding to the actual environment, and not a previous one.

This study requires replication and acknowledges its limitations. The design of the unloading system used in this study may provide some support or restrict movement about the hips in a way that could influence gait. This could have affected participant’s movement strategies in this study. Indeed, two participants actually increased the peak activity of their medial gastrocnemius muscles at the lowest level of load compared to all other levels, contrary to previous works. Likely this has to do with inter-individual differences, but also potentially with the apparatus being used to test these individuals. Future studies should consider examining this phenomenon in more detail. That being said, while this should be kept in mind when interpreting the above results, it is also important to note that all experimental unloading systems have shortcomings such as unloading only the trunk (suspension systems), offering excess inertial resistance (submersion) or enforcing short epochs of study (parabolic flight). In light of this, the AlterG system successfully allows for unloading paradigms to be studied, albeit with its own potential limitations.

This study was a novel use of both zero-dimensional and one-dimensional kinematic and electromyographic analysis. It found that unloading from 100% of normal body weight to 20% elicited distinct electromyographic responses in the medial gastrocnemius, as well as partly in the rectus femoris. Hip, knee, and ankle kinematics were also affected differentially by loading and unloading, especially at 40% of normal body weight. These findings suggest the previous level of gravitational load is an important factor to consider in determining kinematic and electromyographic responses to the current level during loading and unloading below standard g.

## Data Availability

The raw data supporting the conclusion of this article will be made available by the authors, without undue reservation.
